# Mechanisms of the anti-tumor activity of Methyl 2-(-5-fluoro-2-hydroxyphenyl)-1 H-benzo[d]imidazole-5-carboxylate against breast cancer *in vitro* and *in vivo*

**DOI:** 10.18632/oncotarget.16263

**Published:** 2017-03-16

**Authors:** Mohadeseh Hasanpourghadi, Ashok Kumar Pandurangan, Chandrabose Karthikeyan, Piyush Trivedi, Mohd Rais Mustafa

**Affiliations:** ^1^ Department of Pharmacology, Faculty of Medicine, University of Malaya, Kuala Lumpur, 50603, Malaysia; ^2^ School of Pharmaceutical Sciences, Rajiv Gandhi Proudyogiki Vishwavidyalaya, Bhopal, 462033, India

**Keywords:** breast cancer, microtubule targeting agent, mitotic arrest, mitotic slippage, drug resistance

## Abstract

Microtubule Targeting Agents (MTAs) induce cell death through mitotic arrest, preferentially affecting rapidly dividing cancer cells over slowly proliferating normal cells. Previously, we showed that Methyl 2-(−5-fluoro-2-hydroxyphenyl)-1H-benzo[d]imidazole-5-carboxylate (MBIC) acts as a potential MTA. In this study, we demonstrated that MBIC exhibits greater toxicity towards non-aggressive breast cancer cell-line, MCF-7 (IC_50_ = 0.73 ± 0.0 μM) compared to normal fibroblast cell-line, L-cells (IC_50_ = 59.6 ± 2.5 μM). The IC_50_ of MBIC against the aggressive breast cancer cell-line, MDA-MB-231 was 20.4 ± 0.2 μM. We hypothesized that the relatively high resistance of MDA-MB-231 cells to MBIC is associated with p53 mutation. We investigated p53 and three of its downstream proteins: survivin, cyclin dependent kinase (Cdk1) and cyclin B1. Following treatment with MBIC, survivin co-immunoprecipitated with caspases with higher affinity in MDA-MB-231 compared to MCF-7 cells. Furthermore, silencing survivin caused a 4.5-fold increase in sensitivity of MDA-MB-231 cells to MBIC (IC_50_ = 4.4 ± 0.3). In addition, 4 weeks of MBIC administration in MDA-MB-231 cells inoculated BALB/c nude mice resulted in 79.7% reduction of tumor volume compared to the untreated group with no severe sign of toxicity. Our results demonstrated MBIC has multiple anti-tumor actions and could be a potential drug in breast cancer therapy.

## INTRODUCTION

Deregulation of cell cycle leads to cancer, characterized by abnormal chromosome segregation and uncontrolled cell proliferation [1]. The majority of cancer related deaths are due to chemo-resistance of metastatic cells to conventional therapy [2]. Cancer metastasis is a selective cell survival process. Non-metastatic cells are unable to pass one or several steps of the metastatic cascade, while metastatic cells are able to overcome all the steps. These different characteristics may yield different responses to chemotherapeutic drugs [3]. Breast cancer is one of the highly prevalent cancers among women, and is among the top ten cancers worldwide [4, 5]. Breast cancer metastasizes to the liver, lungs, bones and responds poorly to conventional chemotherapy in most cases [6]. Hence, there is a dire need for novel drugs to treat breast cancer with a proper mechanism.

Microtubule Targeting Agents (MTAs) were introduced as anti-tumor agents as early as 1950s. Until recently, it has been postulated that MTAs kill cells only through mitotic arrest [7]. However, several MTAs such as taxanes and epothilones besides stabilizing microtubules, activate spindle assembly checkpoint proteins and suppress cell proliferation [8]. Taxanes are the most widely used MTAs for the treatment of metastatic breast cancer. However, their anti-cancer effects frequently fail due to the resistance of cells to this drug [9]. Recently, we reported that Methyl 2-(5-fluoro-2-hydroxyphenyl)-1*H*-benzo[*d*]imidazole-5-carboxylate (MBIC) is a highly selective MTA that showed greater toxicity toward cancer cells compared to normal cells [10]. In this study, we explored the efficacy of MBIC against aggressive and metastatic human breast cancer cells, MDA-MB-231 cells in both *in vitro* and *in vivo*, along with non-aggressive human breast cancer MCF-7 cells.

Cells contain mitotic proteins that regulate spindle assembly and mitosis, such as survivin, Cdk1 and cyclin B1. Their essential roles begin in G_2_-M phase [11]. MDA-MB-231 cells contain mutated p53 [12]. Survivin which is involved in both apoptosis and mitosis [13], is negatively regulated by wild-type p53 [14]. Under normal circumstances, survivin attachment to caspases avoids caspase-dependent apoptosis [15]. In this study, we examined the roles of p53, survivin and caspases in the development of resistance in MDA-MB-231 cells to MBIC. In addition, we examined the effects of MBIC monotherapy or combination therapy in established human breast tumor xenografts in the BALB/c nude mice. The results provide preclinical evidence that MBIC has potential therapeutic applications in the treatment of aggressive and non-aggressive breast cancer subtypes.

## RESULTS

### MBIC is cytotoxic to breast cancer cells

**Figure 1:**
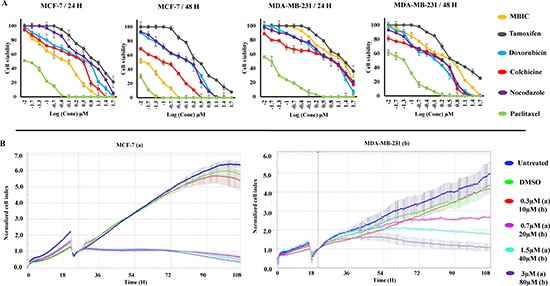
MBIC inhibits MDA-MB-231 and MCF-7 cell proliferation Cells were treated with various concentrations of MBIC or DMSO for 24 and 48 h before determination of (**A**). Cell viability dose-dependently using MTT assay, and (**B**) Cell proliferation time-dependently using RTCA system for 90 h after MBIC application. All results were expressed as total percentage of viable cells of three independent experiments (*P* < 0.05) with mean ± SD.

Cytotoxic effects of MBIC in three chemotherapy-responsive breast cancer cell-lines, MCF-7 (non-aggressive), T47D and MDA-MB-468 (lowly metastatic) [16], intermediate chemotherapy-responsive breast cancer cell-line, MDA-MB-231 (aggressive and highly metastatic) [16] and two normal fibroblast cell-lines, NIH/3T3 and L-cells were assessed by MTT assay. Results showed that IC_50_ of MBIC was 0.73 ± 0.0 μM against MCF-7 cells, 1.3 ± 0.1 μM against T47D cells, 12.0 ± 0.3 μM against MDA-MB-468 cells and 20.4 ± 0.2 μM against MDA-MB-231 cells at 24 h. While, IC_50_ of MBIC-treated NIH/3T3 and L-cells were 55.0 ± 0.1 μM and 59.6 ± 2.5 μM respectively (Table 1). Among selected breast cancer cell-lines, MBIC treatment showed highest toxicity against MCF-7 cells and lowest toxicity against MDA-MB-231 cells. In fact, MDA-MB-231 emulated by over 27-fold more resistance to MBIC. Due to the wide variation in the IC_50_ range, their different p53 status [17] and their different metastatic status [16], MCF-7 and MDA-MB-231 were studied further to identify the underlying molecular mechanism of MBIC. Moreover, the mechanism of resistance of MDA-MB-231 cells was investigated in this study. Further cell viability was measured at 48 h (Table 1). In addition we compared cytotoxic effects of MBIC with a number of conventional anti-cancer drugs, namely, colchicine, nocodazole, paclitaxel, doxorubicin and tamoxifen (Table 1). Figure 1A shows inhibitory effect of MBIC, tamoxifen, doxorubicin, colchicine, nocodazole and paclitaxel against MCF-7 and MDA-MB-231 cells proliferation at 24 and 48 h after treatment.

**Table 1:** Inhibitory effect of MBIC against human cancerous and non-cancer cell-lines

Cell-line	IC_50_ (μM)/24 hours											
	MBIC (MW: 286 g/mol)		Colchicine (MW: 399.43 g/mol)		Nocodazole (MW: 301.3 g/mol)		Paclitaxel (MW: 853.906 g/mol)		Doxorubicin (MW: 543.52 g/mol)		Tamoxifen (MW: 371.51 g/mol)	
	IC_50_	SI	IC_50_	SI	IC_50_	SI	IC_50_	SI	IC_50_	SI	IC_50_	SI
MCF-7	0.73 ± 0.06	81.6	3.28 ± 0.11	3.0	5.12 ± 0.07	5.4	0.01 ± 0.0002	501.0	5.58 ± 1.02	3.1	10.78 ± 0.69	3.1
MDA-MB-231	20.42 ± 0.23	2.9	8.79 ± 0.23	1.1	10.31 ± 0.19	2.7	0.026 ± 0.002	192.6	9.75 ± 0.67	1.7	23.61 ± 0.69	1.4
T47D	1.33 ± 0.18	44.8	0.81 ± 0.01	12.5	1.28 ± 0.23	21.8	0.046 ± 0.01	108.9	0.22 ± 0.07	78.7	7.99 ± 0.16	4.2
MDA-MB-468	12.00 ± 0.31	4.96	0.16 ± 0.07	62.68	4.08 ± 0.52	6.8	3.60 ± 0.41	1.4	2.55 ± 0.54	6.8	14.71 ± 0.84	2.3
NIH/3T3	55.09 ± 0.17	−	8.01 ± 0.62	−	21.94 ± 2.13	−	4.93 ± 0.47	?−	13.51 ± 0.14	−	27.09 ± 1.20	−
L-cell	59.60 ± 2.52		10.03 ± 0.30		28.02 ± 0.39		5.01 ± 0.23		17.33 ± 0.27		34.22 ± 1.91	
**Cell-line**	**IC_50_ (μM)/24 hours**											
	**MBIC (MW: 286 g/mol)**		**Colchicine (MW: 399.43 g/mol)**		**Nocodazole (MW: 301.3 g/mol)**		**Paclitaxel (MW: 853.906 g/mol)**		**Doxorubicin (MW: 543.52 g/mol)**		**Tamoxifen (MW: 371.51 g/mol)**	
	**IC_50_**	**SI**	**IC_50_**	**SI**	**IC_50_**	**SI**	**IC_50_**	**SI**	**IC_50_**	**SI**	**IC_50_**	**SI**
MCF-7	0.02 ± 0.005	526.0	0.12 ± 0.21	29.4	0.97 ± 0.05	7.3	0.004 ± 0.00	502.5	1.48 ± 0.22	2.0	2.51 ± 0.19	5.2
MDA-MB-231	4.07 ± 0.21	2.5	2.09 ± 0.17	1.6	1.55 ± 0.82	4.5	0.07 ± 0.0004	28.7	1.36 ± 0.09	2.2	4.91 ± 0.52	2.6
T47D	0.52 ± 0.08	20.2	0.007 ± 0.001	504.2	0.45 ± 0.05	15.8	0.013 ± 0.003	154.6	0.08 ± 0.002	37.8	6.08 ± 0.92	2.1
MDA-MB-468	4.07 ± 0.93	2.5	0.04 ± 0.002	1.6	1.93 ± 0.16	3.6	0.98 ± 0.11	2.0	0.76 ± 0.05	3.9	6.91 ± 1.03	1.9
NIH/3T3	3.11 ± 0.41	−	2.11 ± 0.92	−	4.54 ± 0.38	−	0.63 ± 0.07	−	3.11 ± 0.04	−	7.46 ± 1.80	−
L-cell	10.52 ± 1.81		3.53 ± 0.83		7.12 ± 0.41		2.01 ± 0.29		3.03 ± 0.01		13.22 ± 1.04	

Human breast cancer cell-lines MCF-7, MDA-MB-231, T47D and MDA-MB-468, mouse fibroblast cell-lines NIH/3T3 and L-cells were treated with DMSO as vehicle control, MBIC, colchicine, nocodazole, paclitaxel, doxorubicin and tamoxifen for 24 and 48 h. Cell viability was determined by MTT assay and by calculation of half maximal inhibitory concentration (IC_50_). Data were mean ± SD of three independent experiments. (*P* < 0.05). Selectivity Index (SI) is calculated by dividing IC_50_ of non-cancer cell-line (L-cells) against IC_50_ of human cancer cell-lines.

### MBIC inhibits breast cancer cell proliferation time-dependently

Whilst MTT assay demonstrated the cytotoxic efficacy of MBIC in a dose-dependent manner we further dynamically monitored changes in cell attachment, proliferation and death. Real Time Cell Analysis (RTCA) assay was performed on MCF-7 cells as shown in Figure 1B. In control and DMSO treated cells, a noticeable constant increase in cell growth can be seen, as reflected by increase in the cell index (CI) values. In MCF-7 cells, treatment with MBIC at > 0.7 μM resulted in significant inhibition of cell growth, while at 0.3 μM the cell growth was not inhibited significantly. After applying 20 and 40 μM of MBIC to MDA-MB-231 cells, a slow inhibition occurred. Cells treated at 80 μM showed a sudden decrease of cell growth, while, at concentration of 10 μM, the MBIC was not toxic to MDA-MB-231 cells (Figure 1B). The time-dependent experiment was performed for about 90 h post administration of MBIC. MDA-MB-231 cells showed a higher resistance to MBIC compared to MCF-7 cells in both dose- and time-dependent manners.

### MBIC induces mitochondrial-caspase-dependent apoptosis

**Figure 2:**
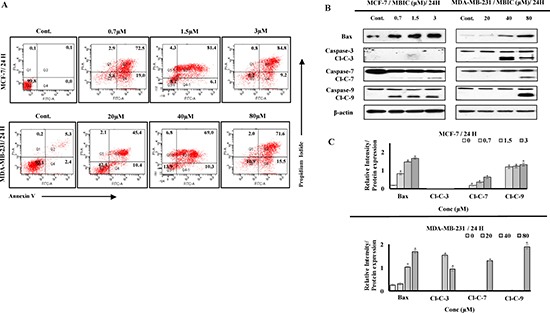
MBIC induces caspase-dependent apoptosis (**A**) Two-dimensional forward and side scatter plots of FITC-conjugated Annexin V vs PI were generated using FACS technology when cells were treated with various concentrations (0.7, 1.5 and 3 μM against MCF-7; 20, 40 and 80 μM against MDA-MB-231) of MBIC for 24 h. Representative figure shows viable cells accumulation (Q3) vs early apoptotic (Q4), late apoptotic (Q2) and necrotic cells (Q1). (**B**) MCF-7 and MDA-MB-231 cells were treated with various concentrations of MBIC (0.7, 1.5 and 3 μM against MCF-7; 20, 40 and 80 μM against MDA-MB-231 cells) before measuring protein level of Bax and cleaved caspases-3/7/9 (Cl-C-3/7/9) with Western blot analysis. β-actin was used as loading control. (**C**) The relative intensity of each protein was normalized with β-actin. Data were results of three independent experiments with mean ± SD. All the treatment groups were compared with control.”*” indicates statistically significant at *P* < 0.05.

Annexin V assay was performed to determine whether MBIC-treated breast cancer cells undergo cell apoptosis. As shown in Figure 2A MBIC treatment of MCF-7 and MDA-MB-231 cells resulted in a higher population of early apoptotic cells (19.0 ± 2.7% to 9.2 ± 3.8% of MCF-7; 10.4 ± 3.5% to 15.5 ± 2.8% of MDA-MB-231) 24 h after treatment compared to the control (0.0 ± 0.1% of MCF-7; 2.4 ± 0.5% of MDA-MB-231). Late apoptosis was observed in both cell-lines (72.5 ± 4.2% to 84.8 ± 2.3% of MCF-7; 45.4 ± 5.2% to 71.6 ± 3.7% of MDA-MB-231) 24 h after MBIC treatment. Our results showed that, MBIC induces apoptotic type of programmed cell death in both breast cancer cell-lines.

**Figure 3:**
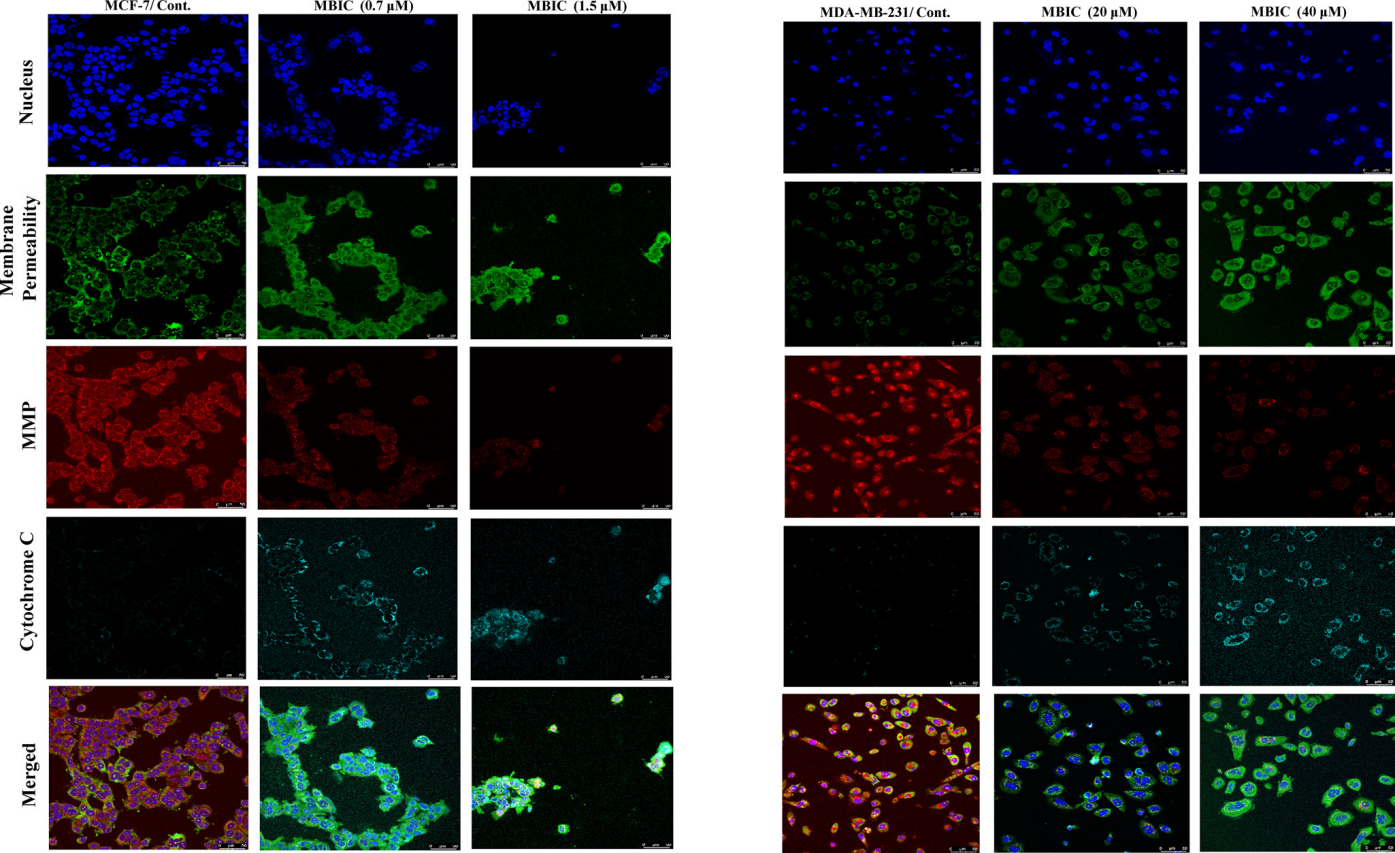
MBIC induces mitochondrial-caspase-dependent apoptosis Effect of MBIC on mitochondrial-caspase-dependent apoptosis in MCF-7 and MDA-MB-231 cells was quantified a using confocal microscope. Cells were treated with various concentrations of MBIC: 0.7 μM (low dose; LD) and 1.5 μM (high dose; HD) against MCF-7, and 20 μM (low dose; LD) and 40 μM (high dose; HD) against MDA-MB-231 for 24 h. Representative figure shows morphological changes by nucleus dye, cell permeability dye, MMP dye and release of cytochrome c by DyLight 649-conjugated secondary antibody probing anti-cytochrome c primary antibody.

To investigate the contribution of pro-apoptotic protein Bax and caspases to the apoptosis, we performed Western blot analysis. As shown in Figure 2B, MBIC induced the cleavage of caspases and elevated the protein level of Bax in both cell-lines. However, activation of caspases in MCF-7 cells is detectable at IC_50_ dosage, unlike in MDA-MB-231 cells (Figure 2B). Figure 2C shows relative intensities of mitotic proteins in a bar graph. After evaluating apoptotic protein levels, mitochondrial-dependent apoptotic features were assessed in MBIC treated cells. MCF-7 cells treated with MBIC after 24 h showed loss of cell population, escalated membrane permeability, and collapsed mitochondrial membrane potential (MMP) compared to control cells (Figure 3). The nucleus in control cells remained in a uniform rounded compact form and size. Permeability dye (green) demonstrated a unified and unbroken plasma membrane. MMP dye (red) was consistently detected in the cytosol, indicating mitochondrial damage in MBIC-treated cells compared to healthy untreated cells. Release of cytochrome c from mitochondria into cytosol was observed (cyan) in MBIC-treated cells but not in untreated cells. In the case of MBIC-treated MDA-MB-231 cells, the average fluorescent intensity of membrane permeability, MMP and cytochrome c release were less than the average fluorescent intensity of these characteristics in MBIC-treated MCF-7 cells, especially in low dosage of MBIC (0.7 μM in MCF-7; 20 μM in MDA-MB-231) (Supplementary Figure 2).

### MBIC arrests cell cycle at G_2_-M phase

To evaluate the effect of MBIC on the cell cycle arrest, the DNA content of both cell-lines was analyzed by flow cytometer. The first peak represents the DNA content of cells that are arrested in the G_0_-G_1_ phase and the second peak represents the DNA content of the cells accumulated in the G_2_-M phase [18], while the rise of third peak is a sign of mitotic exit or mitotic slippage [11].

**Figure 4:**
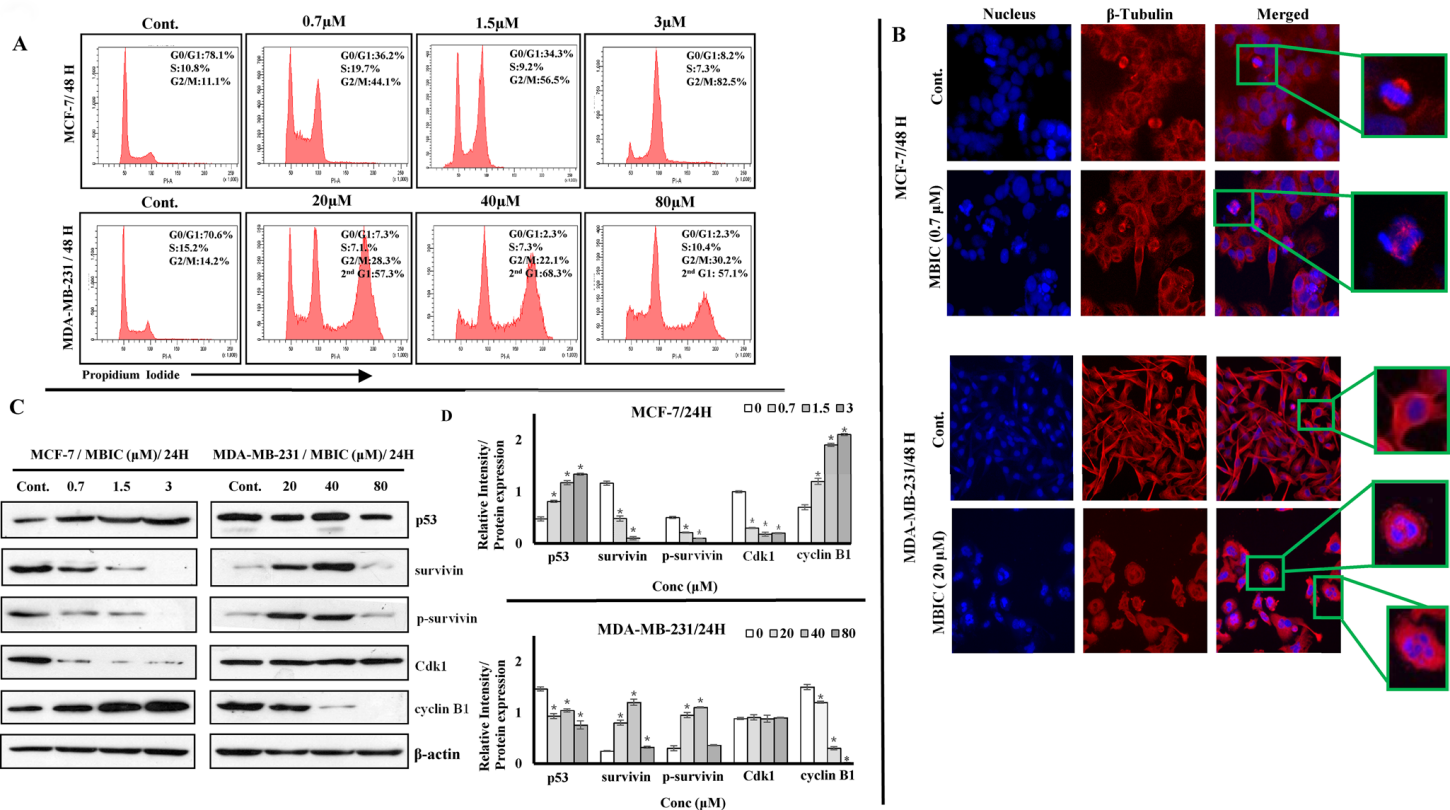
MBIC induces G_2_-M cell cycle arrest in MCF-7 and mitotic slippage in MDA-MB-231 cell-lines (**A**) Flow cytometry analysis of cells treated with various concentrations of MBIC (0.7, 1.5 and 3 μM against MCF-7; 20, 40 and 80 μM against MDA-MB-231) for 48 h was carried out. Representative figure shows distribution of MBIC-treated MDA-MB-231 and MCF-7 cell-lines in different cell cycle phases. Data were results of three independent experiments (*P* < 0.05) with mean ± SD. (**B**) MBIC interrupts cytoskeletal rearrangement: HCS reader with 20 × objective was used. Representative figure shows cytoskeletal β-tubulin rearrangement in MBIC-treated MDA-MB-231 and MCF-7 cells. Cells were fixed, washed, probed with anti-β-tubulin antibody and stained with DyLight 554-conjugated secondary antibody (red) and Hoechst 33342 (blue) to detect β-tubulin and nucleus respectively after treatment with 0.7 μM (against MCF-7) and 20 μM (against MDA-MB-231) of MBIC for 48 h. (**C**) MBIC alters mitotic protein levels: Western blot analysis was done to assess changes of mitotic protein levels. MCF-7 and MDA-MB-231 cell-lines were treated with MBIC dose-dependently (0.7, 1.5 and 3 μM against MCF-7; 20, 40 and 80 μM against MDA-MB-231 cells). (**D**) The relative intensity of each proteins was normalized with β-actin. Data were results of three independent experiments with mean ± SD. All the treatment groups were compared with control.”*” indicates statistically significant at *P* < 0.05.

In both breast cancer cell-lines, treatment with different dosages of MBIC showed G_2_-M phase arrest after 24 h which represents the population of arrested cells at mitosis (Supplementary Figure 1A). Interestingly, 48 h after treatment with MBIC, MDA-MB-231 cells revealed the emergence of a third peak which represents accumulation of cells that escaped mitosis through mitotic slippage and were arrested in a pseudo G_1_/tetraploid state (> 57%). In contrast, MCF-7 cells 48 h after MBIC treatment still showed a higher G_2_-M phase arrested population (44.1 ± 7.3% to 82.5 ± 2.5%), compared to 11.1 ± 3.7% in untreated cells (Figure 4A).

### MBIC interrupts cytoskeleton components

Following the appearance of a third peak in cell cycle detection 48 h post MBIC application, β-tubulin defects were monitored to evaluate cytoskeletal rearrangement. After 48 h of incubation with MBIC, MDA-MB-231 and MCF-7 cells displayed size change, morphological deformation and loss of cell-cell contact sites. Interestingly, the main difference between the two cell-lines appeared to be at IC_50_ dosage of MBIC, as at 0.7 μM of MBIC, MCF-7 cells showed abnormal chromosome segregation and aberrant spindle assembly formation. While at 20 μM of MBIC, MDA-MB-231 cells contained random dispersed and condensed nuclear material throughout the cytoplasm forming multi-nucleated large cells. In contrast to the control cells that contained precise and organized microtubules (Figure 4B). Supplementary Figure 1B shows the cytoskeleton rearrangement at 24 h post MBIC treatment with almost the same morphology as 48 h post MBIC treatment.

### MBIC induces mitotic arrest and mitotic slippage

To identify the underlying molecular mechanism of mitotic arrest and mitotic slippage, the signaling pathway involved in the G_2_-M cell cycle was investigated by Western blot analysis. In this study, facilitative role of Cdk1 and cyclin B1 in cell cycle progression at G_2_-M [19] and survivin's dual role as mitotic effector and apoptosis inhibitor [13], positioned these three proteins in the front line for investigation. In the present study, up-regulation of cyclin B1 was observed in MCF-7 cells after MBIC application, while MDA-MB-231 cells showed down-regulation of cyclin B1. Previously, down-regulation of Cdk1 and survivin was reported to manifest the existence of functional p53 [14, 20]. In the present study, down-regulation of Cdk1 and survivin was observed in MCF-7 cells after MBIC application. While, in MDA-MB-231 cells Cdk1 was stable and survivin was up-regulated, which manifested the existence of dysfunctional p53 [12] (Figure 4C). Figure 4D shows relative intensities of mitotic proteins in a bar graph.

### MBIC alters survivin affinity for caspases

**Figure 5:**
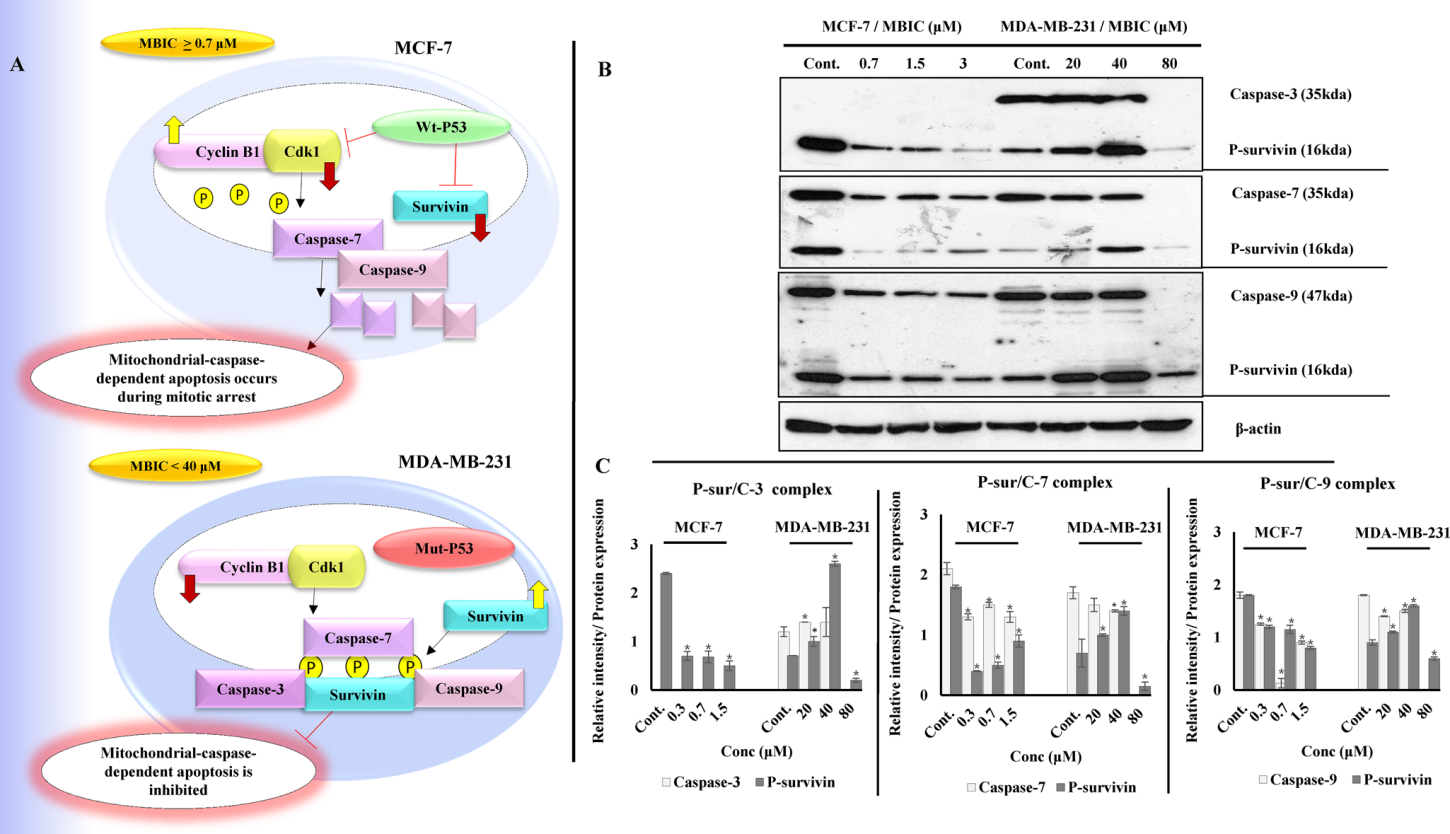
(**A**) Illustration of p53 status and its downstream effect in MCF-7 and MDA-MB-231 cells post MBIC treatment: Functional wild-type p53 in MCF-7 cells suppresses survivin and Cdk1 protein levels after MBIC application. Low protein level of Cdk1 causes lack of phosphorylated survivin. Low protein level of survivin and p-survivin causes release and activation of caspases in cytoplasm which leads to apoptosis (at IC_50_ dosage of MBIC and higher). In contrast, dysfunctional mutated p53 in MDA-MB-231 cells, is not capable of inhibiting survivin and Cdk1 proteins after MBIC application. Therefore, there are plenty of survivin in the cell to be phosphorylated and there are enough Cdk1 to phosphorylate the survivin in Thr34 region. Thus, phosphorylated survivin binds to caspases and inhibits their activation (at dosage above 40 μM). (**B**) MBIC alters affinity of survivin for caspases in MCF-7 and MDA-MB-231 cell-lines with different variations: MDA-MB-231 and MCF-7 cells were treated with MBIC dose-dependently. Complex formation of p-survivin with caspases-3/7/9 was detected while performing Co-IP assay followed by Western blot analysis. (**C**) Bar graph represents dual quantification of p-survivin and caspases-3/7/9 protein intensities in complex formation which are normalized with β-actin. Data were results of three independent experiments with mean ± SD. All the treatment groups were compared with control.”*” indicates statistically significant at *P* < 0.05.

Phosphorylation of survivin at Thr34 residue avoids the activation of caspases and the initiation of caspase-dependent apoptosis [15]. In Figures 4C and 2B, while p-survivin level decreased, caspases-7/9 were activated in MBIC-treated MCF-7 cells. In contrast, while p-survivin level increased in MDA-MB-231 cells, caspases-3/7/9 were only activated in higher dosages of MBIC (40 μM for Caspase-3 and 80 μM for Caspases-7/9). These results suggest p-survivin and caspase status are associated and their association correlates with p53 status. To comprehend this molecular mechanism, Figure 5A illustrates the association between p53 status, survivin and caspases.

To verify Western blot results, a Co-Immunoprecipitation (Co-IP) assay was performed to visualize p-survivin and caspases complex formation. As shown in Figure 5B in MCF-7 cells, along with increase of MBIC dosage, decrease of p-survivin was observed in parallel with a decrease of p-survivin-caspases-7/9 complex. In MDA-MB-231 cells, along with increase of MBIC dosage, there was an increase of p-survivin. This was in parallel with a stable level of p-survivin-caspases-3/7/9 complex at 20 and 40 μM of MBIC and in untreated cells. Finally at 80 μM of MBIC, p-survivin is no longer coupled to caspases. Figure 5C shows dual quantification of p-survivin and caspases-3/7/9 protein intensities in complex formation.

### Gene silencing sensitizes MDA-MB-231 cell-line to MBIC

**Figure 6:**
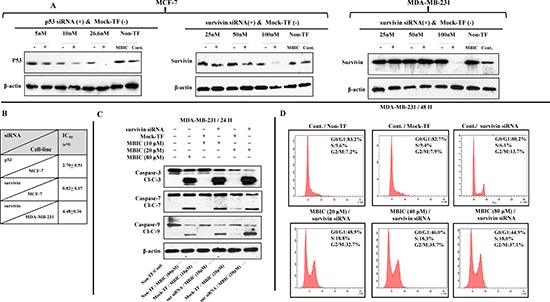
Survivin gene silencing increases the sensitivity of MDA-MB-231 to MBIC (**A**) Cells were transfected with different concentrations of p53 and survivin siRNA, mock-transfection control (Mock-TF) or not transfected control (Non-TF). Western blot analysis was carried out to identify the effective concentration of each siRNA. P53 siRNA at concentration of 26.6 nM succeeded to silence p53 in MCF-7 cells. Survivin siRNA at concentration of 100 nM succeeded to silence survivin in MDA-MB-231 and MCF-7 cells. β-actin served as a loading control. (**B**) IC_50_ of both cell-lines after silencing p53 and survivin. (**C**) Western blot analysis was carried out to evaluate caspases-3/7/9 activation after survivin siRNA transfection in MDA-MB-231 cells. Cells were divided into six groups: (1) Non-TF and untreated, (2) Non-TF and 80 μM (4-fold > IC_50_) of MBIC treated, (3) Mock-TF and 10 μM (2-fold < IC_50_) of MBIC treated, (4) Survivin siRNA transfected and 10 μM of MBIC treated, (5) Mock-TF and 20 μM (= IC_50_) of MBIC treated and (6) Survivin siRNA transfected and 20 μM of MBIC treated. β-actin served as a loading control. (**D**) Cell cycle analysis was performed to investigate the absence or presence of mitotic slippage in MDA-MB-231 cell-line, in four different survivin siRNA transfected groups (with/without MBIC treatment), one Non-TF-untreated group and one Mock-TF-untreated group. Data were results of three independent experiments with mean ± SD.

In order to elucidate the role of p53 in the anti-cancer actions of MBIC, MCF-7 cells were transfected with p53 siRNA. Silencing p53 increased the IC_50_ of MBIC in the MCF-7 cells to 2.7 ± 0.5 μM which represents almost four times less sensitivity to MBIC compared to non-transfected MCF-7 cells. Similarly, survivin was silenced in MCF-7 and MDA-MB-231 cell-lines. In MBIC-treated MCF-7 cells, silencing of survivin was without significant change in IC_50_ (0.82 ± 0.0 μM). However, in survivin siRNA transfected MDA-MB-231 cells, there was a significant decrease of IC_50_ (from 20.4 ± 0.2 μM in non-transfected MDA-MB-231 to 4.4 ± 0.3 μM in siRNA transfected MDA-MB-231). These results suggest that survivin is involved in MDA-MB-231 resistance to MBIC (Figure 6A and 6B).

Further, Western blot analysis was carried out in survivin siRNA transfected MDA-MB-231 cells. Results showed caspases-3/7/9 are activated in the absence of survivin post MBIC application (Figure 6C). Additionally, cell cycle analysis was performed in survivin siRNA transfected MDA-MB-231 cells. Results showed the absence of mitotic slippage 48 h after the treatment of MBIC (Figure 6D).

### MBIC reduces the tumor volume in xenograft mice

**Figure 7:**
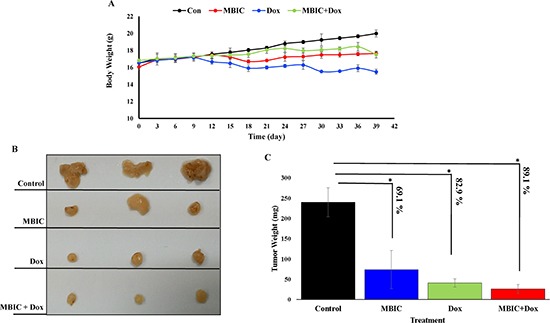
MBIC reduced the tumor growth in xenograft mice (**A**) Body weight before treatment and during 30 days of treatment. (**B**) Represents photograph of tumor sizes after removing from nude mice. We can observe clear reduced size of tumor after treatment with MBIC, doxorubicin and combination treatment (MBIC with doxorubicin) compared to the DMSO-treated group. (**C**) Represents the tumor weights in 4 groups after removal of the tumor from animals. Figure shows 69.1%, 82.9% and 89.1% reduction of tumor weight in MBIC, doxorubicin and combination treatment groups, compared to untreated group respectively. “*” denotes statistical difference (*P* < 0.05).

**Figure 8:**
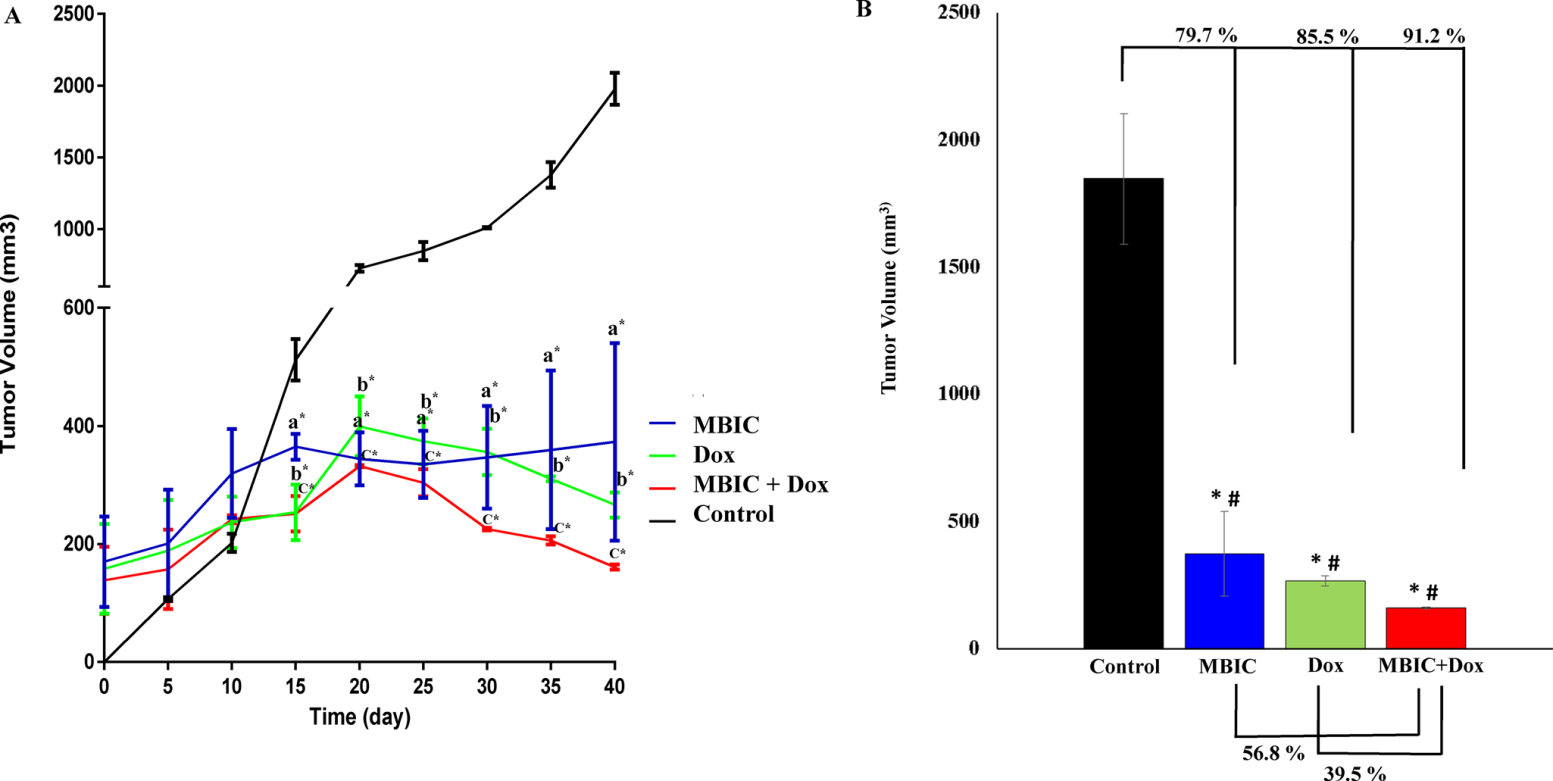
MBIC reduced the tumor volume in xenograft mice (**A**) Reduction of tumor volume in MBIC, doxorubicin and combination treatment groups during 40 days of *in vivo* study. Comparisons: ^a^Tumor Control vs MBIC, ^b^Tumor Control vs doxorubicin, ^c^Tumor Control vs MBIC+doxorubicin. (**B**) 79.7%, 85.5% and 91.2% tumor volume reduction in MBIC, doxorubicin and combination treatment groups, compared to the untreated group. “*” denotes statistical difference (*P* < 0.05) between untreated group vs other groups. Combination of MBIC and doxorubicin shows 39.5% reduction of tumor volume compared to doxorubicin treatment alone, and 56.8% reduction of tumor volume compared to the MBIC treatment group alone. This indicates synergism of MBIC and doxorubicin in combination. “#” denotes statistical differences (*P* < 0.05) between combination group vs MBIC and combination group vs doxorubicin-treated groups.

The *in vivo* anti-tumor effects of MBIC alone and in combination with doxorubicin were evaluated against MDA-MB-231 cells inoculated in BALB/c nude mice. In the present study no severe signs of toxicity were observed, and similarly there was no gross reduction in body weight (Figure 7A). Figure 7B showed the macroscopic analysis of tumor size in untreated and treated groups of mice. MBIC reduced the tumor weight significantly (*P* < 0.05) with the mean tumor weight of 74.0 ± 47.0 mg compared to the untreated tumor-bearing group with mean tumor weight of 240.2 ± 36.1 mg (Figure 7C). Doxorubicin treatment alone reduced the mean MDA-MDB-231 tumor weight to 41.1 ± 6.0 mg. However, following the combined treatment of MBIC and doxorubicin, the tumor weight in the nude mice was further reduced to 26.3 ± 5.0 mg (Figure 7C). In line with the changes in tumor weight, we observed a significant reduction (*p* < 0.05) in the tumor volume in mice treated with a combined treatment of MBIC and doxorubicin (161.2 ± 4.1 mm^3^) compared to either MBIC alone (373.4 ± 167.0 mm^3^) or doxorubicin alone (266.6 ± 11.2 mm^3^). The mean tumor volume of untreated MDA-MDB-231 tumor-bearing group was 1846.6 ± 257.1 mm^3^. Figure 8A shows the tumor reduction between four groups of treatment during 40 days of *in vivo* study. Figure 8B shows tumor volume reduction between four groups of treatment in the last day of *in vivo* study after obtaining the tumors. The present result showed that MBIC actively reduced the tumor volume and weight in MDA-MB-231 cells inoculated xenograft mice, also the anti-tumor effect is even increased when given in combination with the doxorubicin.

## DISCUSSION

Microtubule Targeting Agents (MTAs) are utilized widely in chemotherapy however, most tumors treated with these drugs eventually develop resistance to the agent and continue to proliferate. The limited success of MTAs has led to further investigation and development of more potent and selective drugs [21]. A preliminary report showed that MBIC is cytotoxic to several cancer including breast cancer cells [22]. MBIC exhibits higher toxicity against human cancer and relatively low toxicity against normal cell-lines (IC_50_ > 50 μM), Therefore it is a potential candidate for cancer therapy [23]. In the present study, we evaluated the molecular mechanism of the anti-cancer actions of MBIC against highly aggressive/metastatic and non-aggressive human breast cancer cells. MBIC inhibited cell proliferation with IC_50_ of 0.73 ± 0.0 μM and 20.4 ± 0.2 μM in MCF-7 and MDA-MB-231 cell-lines respectively. MBIC demonstrated relatively low toxicity against two non-tumorigenic cell-lines, NIH/3T3 and L-cells with IC_50_ of 55.0 ± 0.1 μM and 59.6 ± 2.5 μM respectively. The possible reason for greater selectivity of MBIC towards cancer cells compared to normal cells, could be that MBIC interferes with mitotic progression by disrupting the microtubule polymerization. Cancer cells undergo mitosis at a significant increased rate compared to normal cells. This characteristic makes the cancer cells more susceptible to MTAs [24] such as MBIC.

Interruption of microtubule dynamics by MTAs subsequently causes disruption of microtubule-kinetochore binding. Weakly attached or unattached kinetochores send signals to mitotic checkpoint proteins, and in response mitosis is arrested [25]. In the present study, MBIC induced mitotic arrest and mitochondrial mediated apoptosis in both breast cancer cell-lines. Cell cycle analysis showed both breast cancer cell-lines were arrested in G_2_-M phase 24 h after MBIC treatment. However 48 h after treatment, MDA-MB-231 cells showed presence of a third peak (a pseudo-G_1_) [26]. Pseudo-G_1_ state is described as representative of mitotic slippage wherein cells escape a prolonged mitotic arrest, fail cytokinesis and enter into the next G_1_ state of the cell cycle and become tetraploid while still are exposed to MTA [26]. Exit from mitosis requires cyclin B1 degradation by Cdk1, and in case of microtubule disruption, mitotic checkpoints prevent cyclin B1 degradation until entire microtubule-kinetochore connection errors are corrected [27]. However, when microtubule disruption is massive and mitotic checkpoints are not capable of correcting microtubule-kinetochore errors, the cell will then exit mitosis via mitotic slippage by degrading cyclin B1, which is reported to be degraded by many of MTAs [28]. In contrast, cyclin B1 accumulation is generally thought to represent mitotic arrest [29]. In the present study, cyclin B1 was degraded in MBIC-treated MDA-MB-231 cells thereby indicating that cells exited mitosis. In contrast, cyclin B1 was up-regulated in MBIC-treated MCF-7 cells indicating mitotic arrest.

To explain the different sensitivities of MCF-7 and MDA-MB-231 cell-lines towards MBIC, we evaluated the levels of several key proteins. In general, cancer cells with mutated p53 such as MDA-MB-231 cells are reported to display resistance to several MTAs [12, 30]. Survivin is a bi-functional protein that inhibits apoptosis based on inhibitory effect on caspases, and regulates formation of the mitotic spindle during the G_2_-M phase [13]. Loss of wild-type p53 reinforces the up-regulation of survivin in breast cancer, and increase of wild-type p53 suppresses survivin [31, 32]. MBIC dose-dependently decreased the protein level of survivin and increased the protein level of p53 in MCF-7 cells. This finding indicates that p53 is wild-type and remains functional in MCF-7 cells. In MBIC-treated MDA-MB-231 cells MBIC increased protein level of survivin but decreased the protein level of p53. This is related to non-functionality of mutated p53 in this cell-line. In addition, wild-type p53 suppresses Cdk1 expression in the G_2_-M phase and Cdk1 is required for the apoptosis inhibitory role of survivin [33]. In the present study, due to the functional p53, treatment with MBIC decreased the protein level of Cdk1 in MCF-7 cells. In contrast, treatment with MBIC did not affect Cdk1 protein level in the MDA-MB-231 cells and this may be attributed to the dysfunctionality of mutated p53 [33, 34]. On the basis of present data, we can conclude that the reason for mitotic slippage in MDA-MB-231 cells is degradation of cyclin B1 and/or lack of p53 proper function in Cdk1 and survivin regulation. To investigate whether p53 and its downstream proteins contribute to the appearance of mitotic slippage, p53 gene was silenced in MCF-7 cells. The results showed an increase of IC_50_ (3.6-fold), confirming the role of p53 in maintaining sensitivity of MCF-7 cells to MBIC. Next, we silenced the survivin gene in both cell-lines. Significant change was observed in IC_50_ of MDA-MB-231 cells with a 4.5-fold reduction, which confirms that survivin is a causative factor for MDA-MB-231 sensitivity/resistance to MBIC.

Since it was not clear how survivin correlated with observed apoptosis, the association between survivin and caspases was analyzed. Cdk1-cyclin B1 complex phosphorylates survivin at the Thr34 residue on the mitotic apparatus. *In vitro* and *in vivo* studies showed loss of Thr34 phosphorylation results in dissociation of survivin from caspases-3/7/9, activation of caspases and initiation of caspase-dependent apoptosis [15]. In the present study, increased caspases activation coincided with decrease of p-survivin in MBIC-treated MCF-7 cells, while in the contrary, lack of caspase activation coincided with increased level of p-survivin in MBIC-treated MDA-MB-231 cells, except for higher dosage of MBIC in MDA-MB-231 cells. This finding demonstrates a reverse-connection between p-survivin level and caspases activation. Confocal microscopy result confirmed mitochondrial-caspases-dependent apoptosis was initiated in MCF-7 cells at IC_50_ dosage and higher, while it was initiated in MDA-MB-231 cells only at high dosage of MBIC. Co-IP assay demonstrated that MBIC is likely to elicit gain-of-affinity of survivin for caspases-3/7/9 in both cell-lines, but with different variations. After silencing survivin in MBIC-treated MDA-MB-231 cell-line, Western blot analysis result revealed activation of caspases-3/7/9, and cell cycle analysis revealed lack of mitotic slippage. Of note, in the present study the MCF-7 cell-line evidently lacks caspase-3. MCF-7 cell-line obtained from American Type Culture Collection (ATCC) has been reported not to express caspase-3 [35], followed by offering the suggestion that in such cells, caspase-7 may compensate for the loss of caspase-3 in apoptosis induction [36, 35]. In addition to MBIC-induced mitotic arrest in MCF-7 cells, the actions of MBIC also involved mitochondrial-caspase-dependent apoptosis. Consistent with the notion that apoptosis was detected in MDA-MB-231 cells, while caspases were not activated at 20 and 40 μM of MBIC, cells possibly experienced apoptosis in a caspase-independent pathway [37].

In agreement with the *in vitro* findings, MBIC administration inhibited the growth of MDA-MB-231 xenografts in the nude mice. The purpose of using chemotherapeutic drugs in new combination therapies is to improve the chance of adverse effects reduction at minimal doses [40]. Doxorubicin, a DNA-damaging agent (DDA), is one of the first-line chemotherapeutic drugs for breast cancer treatment despite reports of severe side effects [38]. Since MTAs interfere with the translocation of DNA repair proteins from the cytoplasm to the nucleus, thus in MTA and DDA combination therapy, DNA has less chance to be repaired. This synergism between MTA and DDA explains why combination of MTA and DDA are frequently successful in cancer therapy [39]. In present study, we observed a significant (*p* < 0.05) 79.7 % and 91.2% reduction of tumor volume compared to the untreated group, after MBIC monotherapy and MBIC-doxorubicin combination therapy respectively. Successful synergistic reduction of tumor volume in combination therapy with 39.5 % reduction in comparison with the doxorubicin-treated group, and with 56.8 % reduction in comparison with the MBIC-treated group, was significantly (*p* < 0.05) greater than the inhibitory effect of doxorubicin and MBIC alone (Figure 8B). There was no adverse effect on body weight following treatment with MBIC in a monotherapy or in combination with doxorubicin (Figure 7A). The data suggest that MBIC constitutively is likely to be beneficial in the clinical settings either as a monotherapy or in combination with conventional chemotherapeutic agents.

## CONCLUSIONS

In conclusion MBIC, besides perturbing the microtubule assembly, altered mitotic checkpoint proteins. MBIC is more effective in the MCF-7 cell-line, with functionally intact wild-type p53 compared to the MDA-MB-231 cell-line. MDA-MB-231 cells contain defective mutated p53 which is not capable of restoring its normal function in negatively controlling survivin. This may explain the decreased sensitivity of MDA-MB-231 cells to MBIC. The anti-tumor activity of MBIC in the nude mice as a monotherapy or in combination was confirmed without severe signs of toxicity.

## MATERIALS AND METHODS

### Reagents and chemicals

Nocodazole (Cat#M1404), Colchicine (Cat#C9754), Paclitaxel (Cat#T1912) and Tamoxifen (Cat#T5648) were obtained from Sigma-Aldrich (St. Louis, MO, USA). Doxorubicin HCI (Doxorubicin, Adriamycin^®^; Cat#25316-40-9) was purchased from Pfizer, Inc (New York, USA).

### Test material

The synthesis of Methyl 2-(5-fluoro-2-hydroxyphenyl)-1H-benzo[d]imidazole-5-carboxylate has been described previously [22]. Stock solutions of MBIC were maintained in dimethyl sulfoxide (DMSO), protected from light and stored at −20°C for experimental purposes.

### Animals and welfare

The experimental mice were kept in the Animal Experiment Unit (AEU) of the Faculty of Medicine, University of Malaya, Kuala Lumpur, Malaysia. The use of laboratory animals was approved and cared by the Institutional Animal Care and Utilization Committee (IACUC), University of Malaya, Kuala Lumpur, Malaysia (Ethic No. 2015-181103/PHAR/R/AKP). Animals were placed at 25°C ± 2°C in a 12-hour light/12-hour dark cycle in the animal house and fed with standard rodent pellet diet and water *ad libitum*.

### Cell culture

The human breast cancer cell-lines, MCF-7, MDA-MB-231, T47D, MDA-MB-468, mouse fibroblast cell-lines, NIH/3T3 and L-cells were obtained from ATCC (VA, USA). Cells were maintained in RPMI-1640 medium (MCF-7, MDA-MB-231 and T47D), Leibovitz's L-15 medium (MDA-MB-468), and DMEM medium (NIH/3T3 and L-cells). All media were provided from Gibco, Thermo Fisher Scientific (Rockville, MD, USA) and supplemented with 10% fetal bovine serum (FBS) and 10% penicillin/streptomycin. Cells were incubated at 37°C in 5% CO_2_ incubator.

### MTT assay

MTT assay was performed to assess the cytotoxicity of MBIC. Cells were prepared as described previously [10]. Cells were treated with different concentrations of MBIC (0.02, 0.04, 0.09, 0.19, 0.39, 0.78, 1.56, 3.12, 6.25, 12.5, 25, 50 μM) for 24 h and 48 h. DMSO (0.01%) was used as vehicle control. Cytotoxicity of MBIC was assessed as described previously [10], and was expressed as an IC_50_ value. The percentage of viable cells was calculated by the following formula:

$$CellViability(\%):\frac{A_{Sample}-A_{Blank}}{A_{Control}-A_{Blank}}\times \mathrm{100}$$

Each experiment was done in triplicate.

### Real-time cell analysis assay

Cytotoxic effect of MBIC was monitored in a time-dependent manner by using xCELLigence RTCA system (Roche, Manheim, Germany). 50 μl/well of cell-free completed medium was added in 96-well gold-microelectrode array (E-plate) to perform background measurements. 5 × 10^3^ cell/well were seeded and incubated at room temperature for 30 min. The E-plate was incubated in an E-plate 96 RTCA SP station at 37°C in 5 % CO_2_ incubator overnight. Cell adherence, proliferation and spreading were recorded every 5 min and the cell sensor impedance was displayed as an arbitrary unit called normalized Cell Index (nCI). When the cells reached the stage of logarithmic growth, they were treated with various concentrations of MBIC (0.3, 0.7, 1.5 and 3 μM for MCF-7; 10, 20, 40 and 80 μM for MDA-MB-231) and were monitored continuously. To reduce variation between wells, CI values were normalized to the value at the beginning of the treatment time-point. Each experiment was performed in triplicate.

### Apoptosis detection assay

MBIC-induced apoptosis was detected by dual staining Propidium Iodide (PI) and FITC-conjugated Annexin V using Annexin V Apoptosis Detection Kit (BD Pharmingen). This was followed by flow cytometry analysis using Fluorescence-Activated Cell Sorting (FACS) (BD Biosciences, CA, USA) as described previously [10]. Data acquisition of 10,000 events/sample was carried out. Annexin V single positive represented the cells in early apoptotic stage, while cells with both Annexin V and PI positive represented the cells in the last stage of apoptosis, and PI single positive cells represented necrotic. Each experiment was performed in triplicate.

### Multiparameter cytotoxicity assay

MCF-7 and MDA-MB-231 cells were seeded onto 22 × 22 mm cover slips in a 6-well plate, and subsequently were treated with different concentrations of MBIC. After 24 h, cells were fixed with 4% paraformaldehyde and stained using Cellomics Multiparameter Cytotoxicity 3 Kit (#8408102) according to the manufacturer's protocol. This kit includes dyes that represent morphological changes in the nucleus (blue at 405 nm; channel 1), cell membrane permeability (green at 488 nm; channel 2), MMP (red at 543 nm; channel 3) and release of cytochrome c (cyan at 633 nm; channel 4). Cells were incubated with rabbit anti-mouse DyLight 649-conjugated secondary antibody and Hoechst 33342 (Thermo Fisher Scientific, USA). All confocal fluorescence images were acquired using LAS AF software, version 2.6.0.7266 in Leica TCS SP5 II confocal system (Mannheim, Germany) with a HCX PL APO LAMBDA BLUE 40 ×/1.25 oil objective. Data were processed using LAS X software, version 3.0.0. Each experiment was performed in triplicate.

### Cell cycle analysis assay

For the analysis of cell cycle, cells were prepared as described previously [10]. The cells were then treated at different concentration of MBIC for 24 and 48 h. Later, cells were incubated with PI for 30 min. The cells were then collected and prepared for flow cytometry analysis as described previously [10]. The percentage of cells arrested in G_0_-G_1_, S and G_2_-M were obtained using FACS flow cytometry. Each experiment was performed in triplicate.

### Immunofluorescence detection assay (cytoskeletal rearrangement)

The effect of MBIC on one of the microtubule component (β-tubulin) was examined by using Thermo Scientific Cellomics Cytoskeletal rearrangement kit (#8402402). 1 × 10^4^ cells/well were seeded in 96-well plate and treated with IC_50_ concentration of MBIC for 24 and 48 h. Treated cells were stained to detect β-tubulin and nucleus. Images were acquired and analyzed using ArrayScan High Content Screening (HCS) system (Thermo Scientific, USA) with 20 × objective. Each experiment was done in triplicate.

### Western blot analysis

Cells were treated with MBIC in a dose-dependent manner for 24 h. Cells were harvested, lysed and applied for Western blot analysis as described previously [10]. The primary antibodies that were used in this study included: anti-p53, anti-survivin, anti-phospho-survivin (anti-p-survivin), anti-Cdk1, anti-cyclin B1, anti-Bax, anti-caspase-3, anti-caspase-7, anti-caspase-9 (1:1000) (Cell Signaling Technology, USA) and mouse anti-β-actin (1:40000) (Sigma-Aldrich, St. Louis, MO, USA) antibodies. Western blot images were quantified and processed by ImageJ software (NIH, USA). Each experiment was done in triplicate.

### Co-Immunoprecipitation (Co-IP) assay

Co-IP assay is designed to isolate a protein (bait) along with another protein (prey) to visualize that the bait protein is coupled to prey protein in a complex formation. Pierce Co-Immunoprecipitation Kit (Cat#26149, Pierce; Thermo Fisher Scientific, Rockford, IL) was used according to the manufacturer's protocol. 500 μg of lysate was pre-cleared using control agarose resin to minimize non-specific binding. The lysates were then added into columns that contain 10–75 μg immobilized bait protein antibodies (p-survivin) linked to an amine-active resin and incubated overnight at 4°C. The Co-IP was then eluted and analyzed by SDS-PAGE. Caspases-3/7/9 (prey proteins) were applied as primary antibody separately.

### Gene knockdown by transfection of siRNA

For gene knockdown, Signal Silence^®^ p53 siRNA II (#6562) and Signal Silence^®^ survivin siRNA II (#65546) (Cell Signaling Technology (CST), USA) were used. The MCF-7 cells were seeded at a concentration of 5 × 10^4^ cells/well 24 h prior to transfection in RPMI-1640 complete medium in three different 6-well plates (to optimize three different siRNA/Mock-transfected control concentrations). After 24 h the medium was discarded and cells were washed. Further, FBS free media with 5% pen/strep was applied. Three transfection complex (resulting in final concentrations of p53 siRNA/Mock-transfected control: 5 nM, 10 nM, 26.6 nM per well) were prepared. The cells were treated with 100 μl of transfection complex and incubated for 48 h. However, 24 h after transfection half of the free media was removed and an equal volume of the complete media was added. Cells were then washed and lysed using RIPA buffer supplemented with 10 μl protease inhibitor, sodium orthovanadate and PMSF (Santa Cruz Biotech, USA). Further, the samples were evaluated for their silenced p53 gene by Western blot analysis. Additionally, survivin was silenced in both MCF-7 and MDA-MB-231 cell-lines. Three transfection complexes (resulting in final concentrations of survivin siRNA/Mock-transfected control: 25 nM, 50 nM, 100 nM per well) were prepared. Both gene silencing assays were comprised of mock-transfected control siRNA as a positive control. Non-transfected control cells were incubated in parallel.

### Tumor xenograft in nude mice

Female BALB/c athymic nude mice, 4 weeks old, were purchased from BioLASCO Taiwan Co., Ltd., (Taiwan). Mice were allowed to acclimatize to the new condition for 2 weeks prior to experimental manipulations. Mice were handled with aseptic procedures under a laminar flow cabinet in the Animal Experimental Unit (AEU), University of Malaya, Malaysia.

### Induction of human breast cancer cell-line

At the end of the acclimatization period, 2 × 10^6^ of MDA-MB-231 cells in 100 ml of serum-free media + 100 ml of Matrigel^®^ Matrix Cat#354234 (Corning, Bedford, MA, USA) were subcutaneously injected into both the right and left flanks of each animal using a tuberculin syringe and a 21-gauge needle.

### Tumor growth and treatment

To monitor mammary tumor growth, the diameters of the tumors were measured horizontally and vertically. Treatment began when tumors reached an average size of 200 to 300 mm^3^ and this model was considered as an established growing xenograft model. Tumor-bearing mice were divided into the following four groups (3 mice per group): group 1 DMSO-treated (as control group), group 2 MBIC-treated (25 mg/kg in 1x sterile PBS [40]), group 3 doxorubicin-treated (1.5 mg/kg [41]) and group 4 combination of MBIC-doxorubicin-treated (25 mg/kg of MBIC; 1.5 mg/kg of doxorubicin). Treatments were given intraperitoneally every 2 days. Treatments were conducted for 30 days to compare the treatment effects in different groups. Body weight changes and tumor sizes were recorded at regular intervals. Tumor volumes were calculated by the following formula: ½ (Width × Length^2^). The *in vivo* experiments were finalized 40 days after MDA-MB-231 cells injection by overdose of ketamine-xylazine and tumors were collected for further analysis.

### Statistical analysis

The Student's *T* test or one-way ANOVA in Graphpad Prism was performed. A *P* value < 0.05 was considered statistically significant.

## SUPPLEMENTARY MATERIALS FIGURES AND TABLES


