# Supplementary material for: Mechanisms of the anti-tumor activity of Methyl 2-(-5-fluoro-2-hydroxyphenyl)-1 H-benzo[d]imidazole-5-carboxylate against breast cancer in vitro and in vivo
Source: Oncotarget. 2017 Mar 16;8(17):28840–53. doi: 10.18632/oncotarget.16263 (PMC5438696; doi:10.18632/oncotarget.16263)
Supplement: Supplementary file 1 [file oncotarget-08-28840-s001.pdf]

Mechanisms of the anti-tumor activity of Methyl 2-(-5-fluoro-2-hydroxyphenyl)-1 H-benzo[d]imidazole-5-carboxylate against breast cancer *in vitro* and *in vivo*

Supplementary Materials

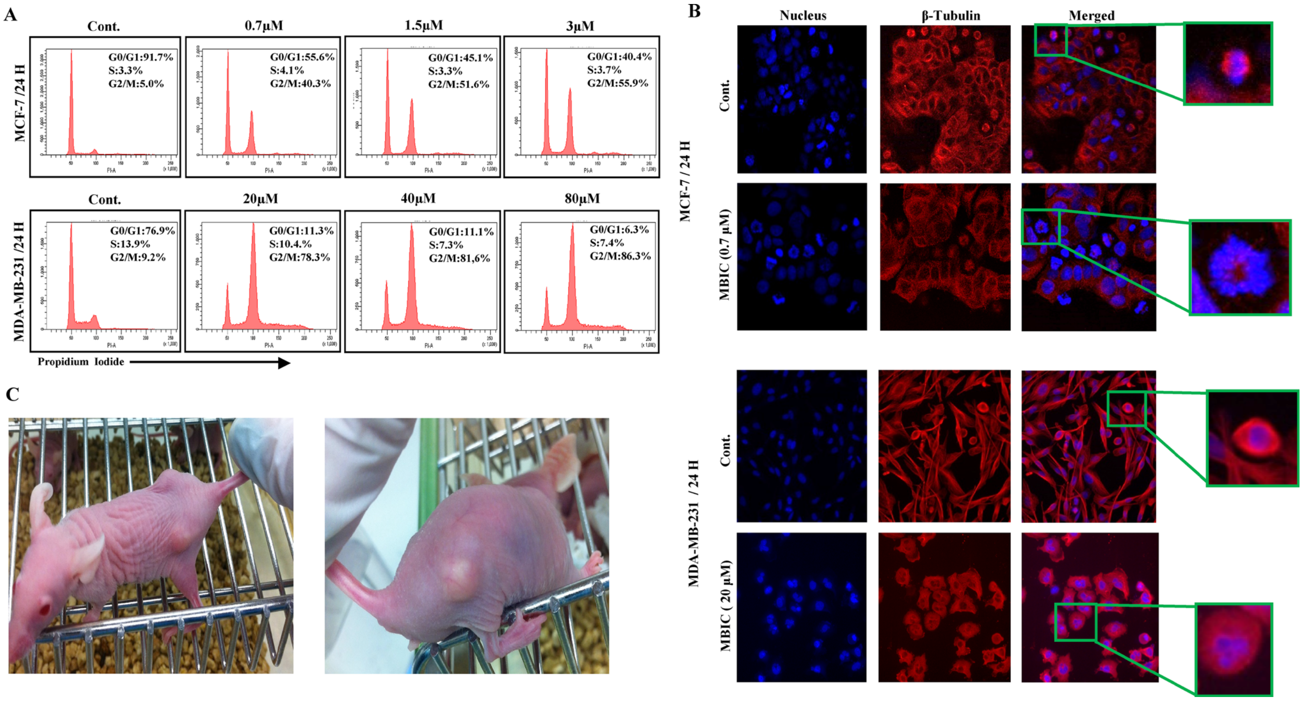

Supplementary Figure 1: (**A**) MBIC induces G2-M cell cycle arrest in MCF-7 and MDA-MB-231 cell-lines 24 h after treatment. Representative figure indicates MBIC arrested both cell-lines in the G_2-M phase 24 h after treatment. Data were results of three independent experiments with mean ± SD. (**B**) Cytoskeleton rearrangements of MCF-7 and MDA-MB-231 cell-lines 24 h after treatment. (**C**) Evidence of tumor growth during 40 days of experiment in tumor-bearing BALB/c nude mice.

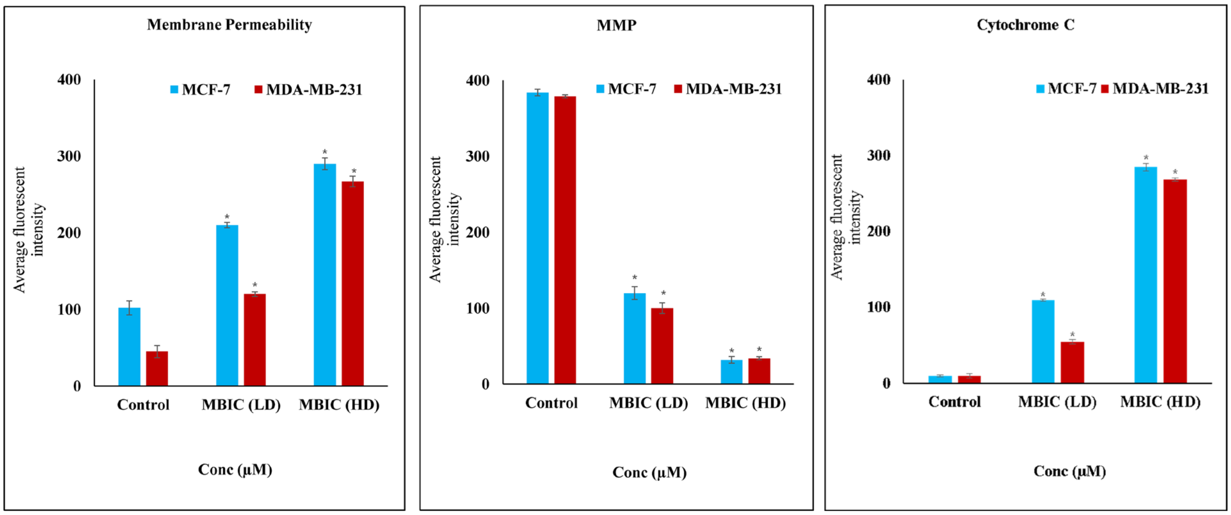

Supplementary Figure 2: Confocal microscope image bar: Bar chart shows different intensities of changes in membrane permeability, MMP and cytochrome c release. Data were results of three independent experiments with mean ± SD. All the treatment groups were compared with control.”*” indicates statistically significant at $P < 0.05$.
